# Evaluating the impact of a modified multi-modal prehabilitation program on perioperative outcomes in Chinese patients undergoing colorectal cancer surgery

**DOI:** 10.3389/fsurg.2026.1703293

**Published:** 2026-02-06

**Authors:** Rui Tai, Fu Yang, Jingyi Wang, Sibei Wan, Qin Xiang, Yuhan Cheng, Fang Fang, Jufang Sun

**Affiliations:** 1 Department of Nursing, Shanghai General Hospital, Shanghai Jiao Tong University School of Medicine, Shanghai, China; 2 Department of Nursing, Shanghai Chest Hospital, Shanghai, China; 3 School of Medicine, Tongji University, Shanghai, China; 4 School of Nursing, Jinggangshan University, Ji’an, Jiangxi, China; 5 Department of Nursing, Shanghai Tenth People’s Hospital, Tongji University, Shanghai, China

**Keywords:** 6-Minute Walk Test (6MWT), colorectal cancer (CRC), Enhanced Recovery After Surgery (ERAS), multimodal interventions, perioperative care, postoperative outcomes, prehabilitation

## Abstract

**Aim:**

This study aims to evaluate the feasibility and effectiveness of a modified multi-modal prehabilitation program for Chinese patients with colorectal cancer during their preoperative hospital stay. The impact on perioperative physiological function, postoperative recovery, and overall outcomes was assessed.

**Design:**

A prospective, randomized controlled trial.

**Methods:**

A prospective, randomized controlled trial was conducted with 200 patients (100 experimental, 100 control) at a tertiary hospital in Shanghai. The experimental group received a modified multi-modal prehabilitation program, including inspiratory muscle training, aerobic exercise, nutritional supplementation, and psychological support, while the control group received standard perioperative care. Primary outcomes were assessed using the 6-Minute Walk Test (6MWT) on the day before surgery and 30 days postoperatively. Secondary outcomes included postoperative hospital stay duration, time to first flatus, ambulation, oral intake, and incidence of postoperative complications.

**Results:**

The experimental group showed significantly greater improvements in 6MWT performance compared to the control group both before surgery (400.40 m vs. 383.25 m, *P* < 0.01) and 30 days postoperatively (375.40 m vs. 336.85 m, *P* = 0.03). Additionally, the experimental group had a shorter postoperative hospital stay (7.91 days vs. 9.06 days, *P* < 0.01) and earlier recovery milestones (*P* ≤ 0.01) compared to the control group. The incidence of postoperative complications was slightly lower in the experimental group, though not statistically significant.

**Clinical Trial Registration:**

https://www.chictr.org.cn/, Identifier ChiCTR2200055764.

## 1 Introduction

Colorectal cancer (CRC) is among the most prevalent malignant neoplasms worldwide. Globally, CRC is the third most frequently diagnosed cancer after lung and female breast cancer, and it ranks second in cancer-related mortality. These figures underscore the substantial public health burden posed by CRC, while also highlighting the continued centrality of surgical treatment for localized disease (1, 2).

Surgical intervention, however, triggers a pronounced perioperative stress response encompassing both psychological and physiological domains, including tissue trauma, anesthesia, pain, and postoperative complications. In this context, Enhanced Recovery After Surgery (ERAS) was developed to optimize perioperative pathways through evidence-based measures that mitigate stress, shorten hospitalization, and reduce costs—benefits that have been demonstrated across diverse surgical populations over the past two decades (3, 4).

Traditional efforts to accelerate recovery have focused predominantly on the postoperative period. Yet patients who present with preoperative frailty, malnutrition, or sarcopenia frequently exhibit reduced physiological reserve and physical performance, which are associated with prolonged length of stay, higher complication rates, and increased mortality (5–7). Mounting evidence indicates that preparing patients before the surgical insult—rather than rehabilitating after it—can foster faster and more durable recovery (8–10). In this vein, “prehabilitation” is conceptualized as a continuous preoperative care process—typically spanning from diagnosis to the day of surgery—aimed at improving tolerance to anticipated surgical stress and enhancing postoperative recovery (11–13).

Despite growing interest, no universally accepted definition, preferred protocol(s), or recommended duration of prehabilitation exists. Common elements include exercise training, nutritional support, and strategies to reduce anxiety, with reported program lengths typically ranging from two to six weeks (14, 15). Real-world adherence to longer regimens can be challenging, and concerns persist regarding potential delays to definitive surgery.

Across many health systems, the preoperative window is brief and constrained by scheduling logistics and patients' preference for timely surgery, making longer prehabilitation programs difficult to implement and raising concerns about delaying definitive treatment. Therefore, we evaluated the feasibility and effectiveness of a short, supervised, inpatient multimodal prehabilitation program before elective colorectal cancer surgery, and examined its impact on perioperative function, postoperative recovery, and overall outcomes within contemporary ERAS pathways.

## 2 Methods

### 2.1 Study design

A prospective randomized controlled clinical trial with two parallel groups was conducted in a tertiary comprehensive hospital from June to December 2023 in Shanghai, China. Patients were recruited during outpatient visits and randomly divided into a control group and an experimental group after the decision of surgery was made by both the surgeon and the patients. This randomized clinical trial was conducted with approval from the ethics committee of the hospital institutional review board, and it was registered on the Chinese Clinical Trial Registry (ChiCTR2200055764) on the 19th January 2022.

### 2.2 Participant recruitment

Recruitment flyers for this study were distributed in the gastrointestinal surgery clinic of the hospital. The researcher met the patients who met all the inclusion criteria and none of the exclusion criteria before hospitalization, explained the research, and provided the participant information statement. The potential participants were given one day to consider. Written informed consent was obtained from all participants before they were enrolled.

Participants were scheduled for elective resection of none-metastatic colorectal cancer; Chinese male or female patients over 18 years old and under 80 years old; Prior written informed consent must be obtained before any assessment. Patients were excluded if they had: co-morbid medical, physical, and mental conditions that contraindicate physical exercise or oral nutrition (e.g., unstable angina or symptomatic severe aortic stenosis), disabled orthopedic and neuromuscular disease, dementia, psychosis; severe cardiac abnormalities, severe end-organ disease such as cardiac failure [class II or above heart failure according to the New York Heart Association (16)], sepsis, severe liver or kidney failure; unable to swallow, or being fed through tube feeding; poor Chinese comprehension.

### 2.3 Randomization

Participants were randomly divided into one of the two study arms by a person who was not a member of the research team using a random allocation sequence generated by Software SPSS 24.0. Allocations were placed in sealed, opaque, consecutively numbered envelopes by an independent researcher. On the day of the patient admission, a nurse in the hospital who has been trained but has no direct caring relationship with the participants, conducted a baseline assessment of the participant. The researcher then opened the next sealed envelope and assigned the participant to the indicated group.

### 2.4 Blinding

Due to the nature of the intervention, blinding was not possible for both the patients and the intervenor. To reduce performance bias, patients were told that we had compared two perioperative intervention plans, but we were unsure which was superior.

### 2.5 Sample size

The sample size for this trial was estimated based on literature (17) and our own unpublished data. We assumed that the average 6-minute walk distance (6MWD) in the experimental group would be 42 m higher than that in the control group at the day before surgery. The SD for 6MWD would be 60 m in both groups. A sample size of 200 (100 per group) was required to detect a statistically significant difference at a 2-sided significance level of 0.05% and 80% statistical power. To account for patient dropouts and missing data, we planned to recruit a total of 280 patients.

### 2.6 Interventions

The intervention commenced immediately after hospital admission. The control group received standard perioperative care. This group received general instructions on nutritional counselling and exercises (deep breathing, effective cough) during the hospital stay by a nurse. The experimental group attended a modified multi-modal prehabilitation program prior to surgery supervised by a multidisciplinary team in the hospital. The modified multi-modal prehabilitation program was designed by the research group based on previous systematic review (18) and evidence summary (19). Preoperative rehabilitation is carried out during the preoperative hospital stay and usually lasts for 7 days. This study systematically monitored and recorded each link of the preoperative rehabilitation intervention.

### 2.7 Perioperative care

The control group received routine perioperative guidance according to the 2018 Guidelines for Perioperative Care in Elective Colorectal Surgery (20). This group received preoperative guidance including routine admission education, nursing risk assessment, nutritional risk screening, cardiopulmonary function assessment, nutritional counseling, anemia correction, postoperative exercise guidance (deep breathing, effective cough). Depending on the patients' postoperative condition, early oral feeding and ambulatory activities should be carried out. Discharge instructions included guidance on diet, medication, and activities, and informed the outpatient follow-up time was 30 days after surgery.

### 2.8 Prehabilitation program

Based on the routine enhanced recovery intervention in the control group, modified multi-modal prehabilitation intervention was implemented. To ensure the quality of intervention, a multidisciplinary team of 8 members was set up, including a head of the nursing department and a director of the gastrointestinal surgery department who were responsible for research design and work assignment, two nurses who were responsible for data collection and follow-up, a gastrointestinal surgeon, a registered dietitian, a psychological consultant and a kinesiologist. All the interventions were implemented by the members of multidisciplinary team.

#### 2.8.1 Exercise training

##### 2.8.1.1 Inspiratory muscle training (IMT)

On the day of admission, patients in the experimental group received one session on IMT and they were supervised to perform this exercise every day at the hospital prior to surgery. Patients were shown how to use the Carent Respiratory Exerciser as an inspiratory muscle training tool. The inspiratory muscles training duration was 15 min. Patients were supervised by the nurses and completed the IMT three times a day.

##### 2.8.1.2 Aerobic exercise training

In addition to the IMT, patients attended an aerobic exercise training session once a day supervised by the kinesiologist at the hospital rehabilitation unit prior to surgery. Aerobic exercise training was completed on a stationary bicycle (LGT-5100, Guangzhou Longest Inc), starting with minimum resistance and gradually increasing according to the patient's heart rate. The aerobic exercise training duration was 30 min with a heart rate of 60%–80% of the patient's maximal heart rate (maximal heart rate = 220- age).

#### 2.8.2 Nutritional supplementation

Malnutrition is common in patients with colorectal malignancies, where the prevalence of malnutrition is between 20% and 70% (21). Studies (22, 23) have shown that malnutrition is an independent risk factor for postoperative complications in patients with gastrointestinal tumors. Therefore, the primary goal of perioperative nutritional therapy was to optimize preoperative nutritional storage and provide sufficient nutrition to compensate for postoperative catabolic response.

The nutritional screening of all participants was implemented at their baseline appointment. Then, both study groups participated in the same counselling session (45 min total) provided by the registered dietitian. During this session, patients were provided with written dietary advice and viewed with a presentation on preoperative nutrition, emphasizing the importance of avoiding unintentional weight loss and increasing protein intake to maintain muscle mass prior to surgery.

Patients in the experimental group were provided with an oral nutritional supplement (Nutrison, Milupa GmbH Inc.) 1,000 ml/d (3.28 Kj, 0.03 g of protein per ml) in addition to their normal diets. Patients were instructed to use these supplements within 1 h after exercise training or before bedtime to maximize muscle protein synthesis. Patients in the control group did not receive additional nutritional supplements beyond their normal diet. Two nurses were responsible for monitoring the patients' preoperative oral compliance and recording the patients' daily oral dose.

#### 2.8.3 Psychological support

As might be expected, patients undergoing colorectal surgery are anxious and fearful. All participants received a psychological assessment by the Hospital Anxiety and Depression Scale (HADS) at the day of admission. If the assessment resulted in a high score (Anxiety Scale of 8 or higher, Depression Scale of 8 or higher), patients were considered high-risk and offered a referral to a psychologist.

Patients in the experimental group received an extra booklet about the clinical pathway of colorectal surgery during the preoperative period by the Department of Surgery. Many pictures and photos were used in the booklet to explain various medical and nursing measures during the perioperative period, such as preoperative examination, prehabilitation and early postoperative rehabilitation. Meanwhile, patients could also scan the QR code in the booklet to watch the relevant video. The possible underlying causes of pain, fatigue and anxiety during the perioperative period and the coping skills were also discussed. In addition, patients received a log where all activities related to prehabilitation will be recorded, and the nurses provided adequate health education to the patients to eliminate the patients' misunderstanding.

Under the guidance of the psychological consultant, patients engaged in deep breathing, meditation, and muscle relaxation activities once a day, for 20 min each session.

### 2.9 Outcomes measures

#### 2.9.1 Primary effect measure

The 6-min walk test (6MWT) (24) was used to evaluate the functional capacity of the body; the data measured on the day of admission were used as baseline indicators. This assessment was conducted on the day of admission, the day before surgery, and the 30th day after surgery. A change of less than ±20 m in 6MWT relative to the baseline indicated equal walking capacity, while an increase of more than 20 m relative to the baseline indicated progress; similarly, a decrease of more than 20 m relative to the baseline indicated decline (25).

#### 2.9.2 Secondary effect measures

The length of postoperative hospitalization, the time to first flatus, the time to first ambulation, the time to first oral intake and drainage removal time were recorded. In addition, we also counted the incidence of postoperative complications in patients within 30 days after surgery (according to the Clavien-Dindo classification 26).

### 2.10 Statistical analysis

Data analyses were conducted using IBM®SPSS Statistics version 26.0 by two people. Shapiro Wilk test was used to perform normality test. Continuous variables that satisfied normal distribution were presented as the means with standard deviations (SDs) and compared by Students *T*-test; variables of non-normal distribution were described by medians with interquartile ranges (IQRs) and compared using Mann Whitney *U*-test; categorical variables were presented in frequencies with percentages and compared by Chi-square test. *P* < 0.05 indicates that the difference is statistically significant.

## 3 Results

**Figure 1:**
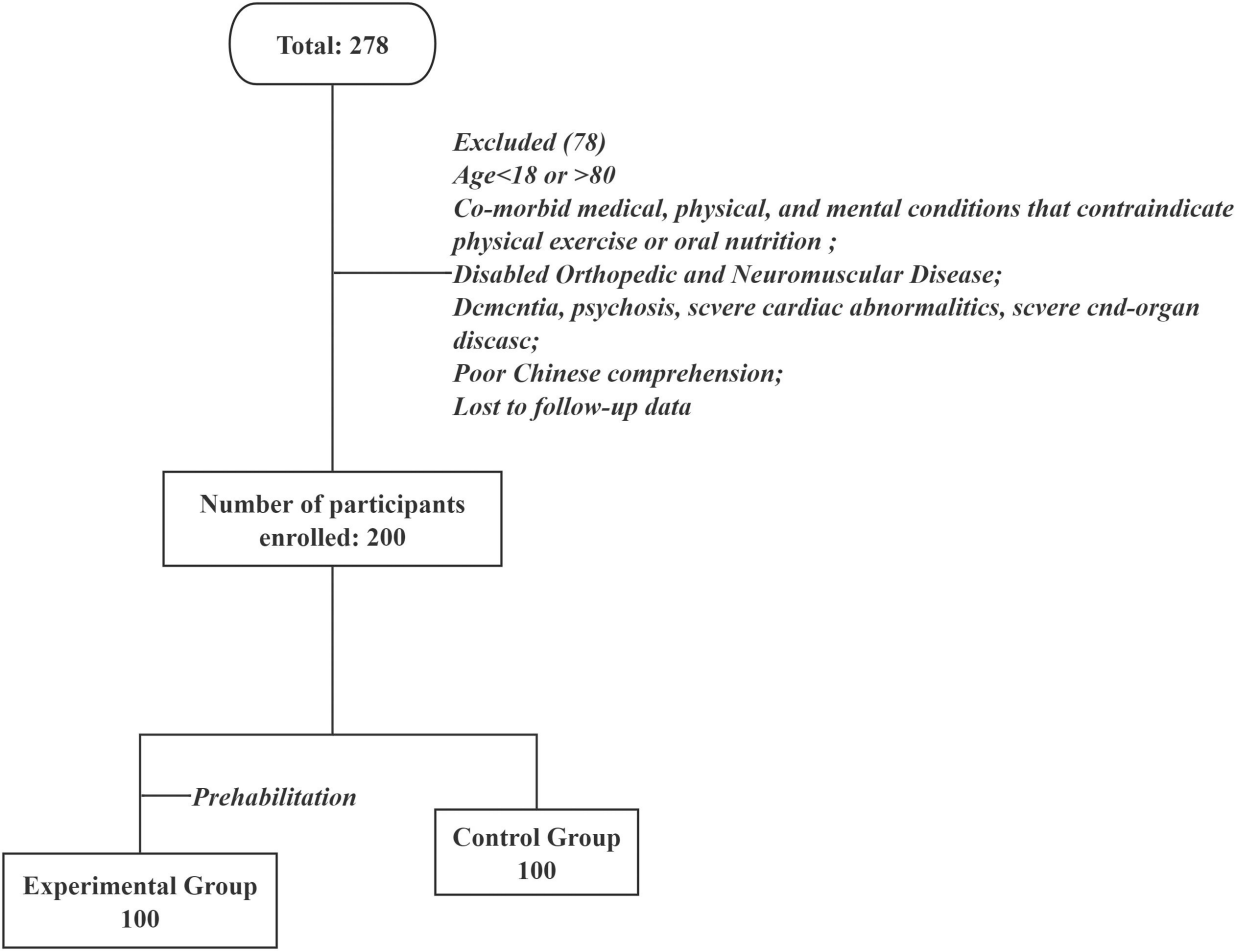
Overview of the study cohort.

The experimental and control groups comprised 100 and 100 patients, respectively. All patients in the intervention group completed the modified multi-modal prehabilitation programmed with no adverse events. Among 278 patients, 200 were included in final analysis, 100 (50%) patients were labelled as experimental group while 100 (50%) as control group (Figure 1). Baseline characteristics of patients are provided in Table 1.

**Table 1:** Basal patient characteristics according to prehabilitation.

Variable	Experimental group (*N* = 100)	Control group (*N* = 100)	*P*-value
Age, years	64.7 [74; 46]	66.5 [77; 34]	0.17
Gender, male (%)	43	51	0.26
BMI (kg/m^2^)	22.86 [36.19; 16.94]	23.09 [30.12; 18.22]	0.59
ASA, Ⅱ (%)	98	99	0.56
Surgical approach (*n*)			
Laparoscopic	82	74	0.25
Open	18	26	
Tumor location (*n*)			
Colon	61	67	0.40
Rectum	39	33	
Stoma formed (*n*)	16	19	0.71
Tumor stage (*n*)			
I	12	15	0.68
II	50	52	
III	38	33	
HADS score			
SD	10.84 [17; 6]	10.56 [18; 1]	0.41
The 30th day after surgery	6.6 [13; 1]	11.42 [16; 5]	<0.01
6MWT (m)			
SD	401.60 [500; 350]	391.40 [500; 320]	0.04
The day before surgery	400.40 [510; 330]	383.25 [500; 300]	<0.01
The 30th day after surgery	375.40 [475; 300]	336.85 [420; 245]	0.03
First flatus (days)	2.17 [4; 1]	2.78 [5; 1]	<0.01
First ambulation (days)	2.53 [4; 1]	3.04 [5; 1]	<0.01
First oral intake (days)	2.30 [4; 1]	2.34 [5; 1]	0.80
The length of postoperative hospital stay (days)	7.91 [12; 6]	9.06 [16; 6]	<0.01
Drainage tube (days)	7.28 [15; 5]	8.00 [15; 5]	0.01

### 3.1 Primary outcomes

The day before surgery, the experimental group exhibited a significantly greater improvement in the 6MWT compared to the control group (400.40 m vs. 383.25 m, *P* < 0.01). The change from baseline in the 6MWT was also markedly better in the experimental group than in the control group (−1.25 vs. −7.95, *P* < 0.01). Additionally, 95% of patients in the experimental group experienced a change of less than ±20 m in the 6MWT relative to baseline, indicating stable walking capacity; within this group, 38 patients showed an improvement in walking ability. Conversely, 58 patients in the control group experienced a decline in walking ability.

**Figure 2:**
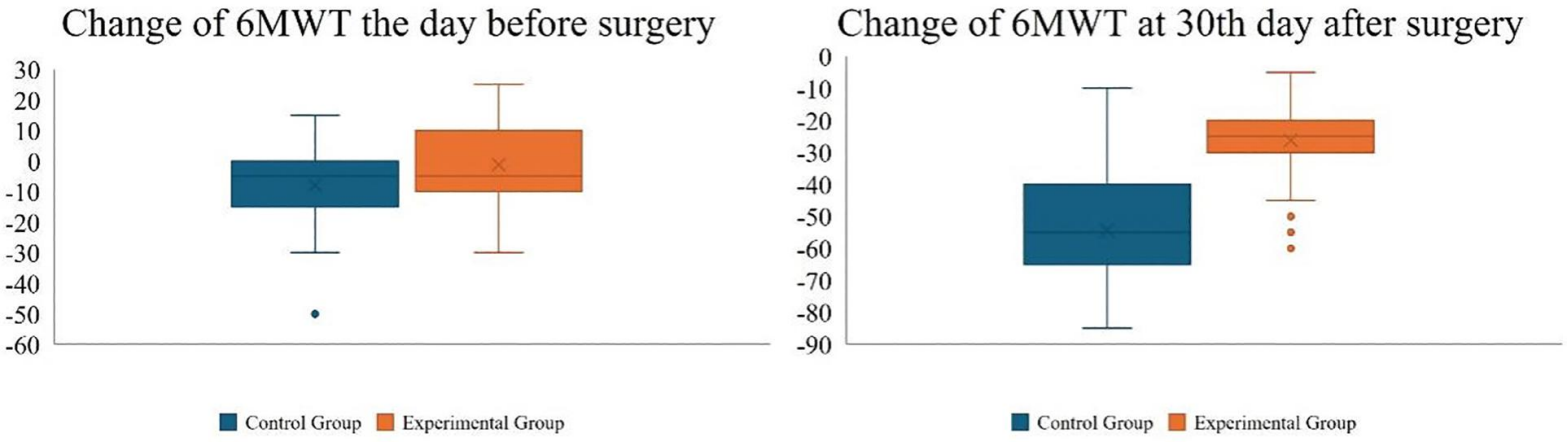
The change from baseline in the 6MWT.

Thirty days postoperatively, both the experimental and control groups showed a decline in 6MWT performance compared to the day before surgery (−26.20 vs. −54.35, *P* < 0.01). However, the experimental group maintained superior walking ability relative to the control group (375.40 m vs. 336.85 m, *P* = 0.03). Compared to baseline, 42 patients in the experimental group returned to their pre-admission walking capacity, whereas 95 patients in the control group exhibited a decrease in walking capacity (Figure 2). All patients in the intervention group completed the established pre-rehabilitation program. The specific compliance situation is as follows: IMT was carried out under the supervision of nurses, with a completion rate of 100%. The daily attendance rate for aerobic exercise is 98.5%. The average daily intake of nutritional supplements reached 96% of the prescribed amount. The participation rate of psychological-related activities (including relaxation exercises and health education) was 94%.

### 3.2 Secondary outcomes

**Figure 3:**
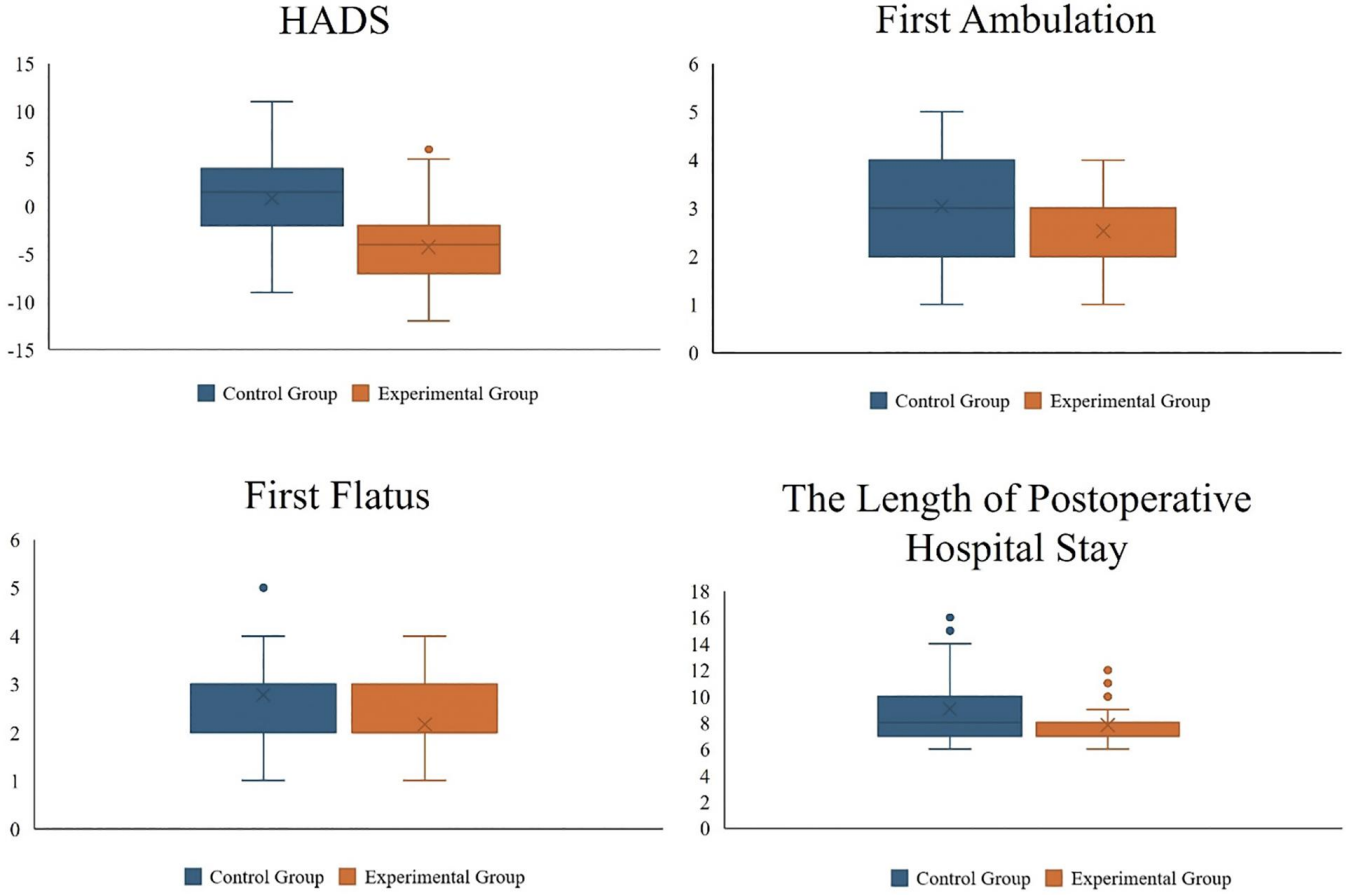
Secondary conclusions drawn from comparing the experimental group with the control group.

Patients in the experimental group were earlier to first flatus, first ambulation, and drainage tube (*P* ≤ 0.01). Notably, the postoperative hospital stay was significantly shorter in the experimental group compared to the control group (7.91 days vs. 9.06 days, *P* < 0.01). This finding suggests that prehabilitation not only improves the 6MWT but also further shortens the postoperative recovery time for patients. Although there was no statistically significant difference between the two groups in the timing of the first oral intake after surgery, patients in the experimental group resumed oral intake slightly earlier than those in the control group. The control group had 20 postoperative complications (Grade I: 12, Grade II: 6, Grade ≥ III: 2), including twelve chyle leakage, six Ileus, and two hemorrhage. The experimental group had 11 postoperative complications (Grade I: 6, Grade II: 5), including six chyle leakage, three anastomotic fistula and two Ileus. There was no significant difference in the incidence of postoperative complications in patients within 30 days after surgery between the two groups of patients (Figure 3).

## 4 Discussion

### 4.1 The impact of preoperative prehabilitation on postoperative functional recovery in patients with colorectal cancer

In this study, a short-term, multimodal preoperative intervention substantially improved the perioperative functional trajectory. Compared with the control group, the intervention group increased 6MWT distances by approximately 17 m on the day before surgery and 39 m at 30 days postoperatively, while postoperative length of stay was shortened by about 1.15 days. Importantly, rehabilitation milestones—including first flatus, first ambulation, and drain removal—were achieved earlier, and the 30-day complication rate did not increase. These findings indicate that even within a limited preoperative window, a standardized prehabilitation program can yield clinically relevant gains in functional recovery and care efficiency without compromising safety.

The improvement was not only statistically significant but also clinically meaningful. Prior studies have established the 6MWT as a reliable functional indicator during recovery from colorectal surgery, with changes reflecting differences in physiological reserve and longer-term prognosis (27). Multiple trials have adopted a ≥20 m change as a clinically relevant threshold; the 39 m between-group difference observed at postoperative day 30 in the present study exceeds that benchmark, suggesting that the functional gains are likely to translate into better mobility and greater independence in daily life (8, 28). These observations are broadly consistent with randomized evidence. Minnella et al. reported that patients receiving prehabilitation demonstrated superior functional recovery both preoperatively and at follow-up, showing greater stability than rehabilitation alone (29). Molenaar et al. further showed that incorporating supervised, multimodal components into prehabilitation can amplify functional benefits (30). In addition, recent systematic reviews indicate that exercise-based or multimodal prehabilitation generally improves functional metrics and, in some studies, shortens hospital stay, although effects on postoperative complications are heterogeneous (31). In line with these data, the present study likewise demonstrates concurrent functional improvement and shorter hospitalization without an increase in complications. Notably, malnutrition and sarcopenia are common in patients with colorectal cancer; limited physiological reserve may heighten responsiveness to preoperative interventions, offering a plausible explanation for the clear benefits observed with short, intensive programs in this population (32).

Taken together, existing evidence and the current results suggest that preoperative prehabilitation can enhance physiological reserve within a constrained timeframe and deliver dual benefits—improved postoperative function and greater inpatient efficiency—without compromising safety. For patient groups in which malnutrition and low fitness are prevalent, such as those undergoing colorectal surgery, this strategy appears particularly valuable. Crucially, the observed functional gains are unlikely to be driven by any single component; rather, they likely reflect the combined influence of respiratory and exercise training, nutritional supplementation, and psychological adjustment acting through complementary physiological and behavioral pathways to amplify overall effects.

### 4.2 Mechanistic interplay of exercise, nutrition, and psychological modulation in prehabilitation

Within perioperative optimization strategies, the superiority of multimodal programs has become increasingly evident. The present findings indicate that improvements in functional recovery and care efficiency are not attributable to any single component but arise from the combined action of exercise training, nutritional supplementation, and psychological modulation. Isolated respiratory or aerobic training can enhance cardiorespiratory reserve; however, without adequate nutritional support its adaptive effects are often constrained (33). Likewise, nutrition given in the absence of exercise provides limited anabolic gain (34). Psychological interventions mitigate anxiety, strengthen adherence, and improve sleep quality, thereby creating the conditions necessary for consistent implementation of exercise and dietary plans (35). Prior randomized trials reinforce this view: for example, Bousquet-Dion et al. showed that supervised, multimodal prehabilitation yields larger functional gains than exercise alone (15), and Mima et al. reported in upper gastrointestinal cancer that combining exercise with nutrition significantly improves postoperative functional capacity (36). Taken together, these data—aligned with the results of the present study—suggest that a multi-target approach accelerates recovery through converging pathways of enhanced physiological reserve, optimized nutrient utilization, and improved behavioral adherence.

At the mechanistic level, respiratory and aerobic training strengthen the diaphragm and accessory inspiratory muscles, improve alveolar ventilation and oxygenation, and thereby lower the risk of postoperative atelectasis and respiratory complications (37). These physiological adaptations not only elevate baseline exercise tolerance but also prime the system for more effective use of nutritional inputs. Evidence supports a post-exercise “window of synergy”, during which anabolic sensitivity is heightened; timely provision of high-quality protein or amino acids promotes myofibrillar protein synthesis and attenuates catabolism, improving perioperative muscle mass and functional reserve (38). Consistent with this concept, the randomized trial by Minnella et al. demonstrated that coupling nutrition with exercise confers greater functional benefit than exercise training alone in upper gastrointestinal cohorts (25).

Psychological modulation is likewise indispensable in this chain. Anxiety and depressive symptoms are common in patients undergoing major abdominal surgery and can amplify stress responses via sympathetic activation and hypothalamic–pituitary–adrenal (HPA) axis upregulation, with downstream immunosuppression and metabolic dysregulation (39). Interventions such as relaxation training and cognitive reframing reduce anxiety, directly improving psychological status and indirectly enhancing adherence to prescribed exercise and dietary regimens. In a randomized trial, Bausys et al. showed that embedding structured psychological and behavioral support within prehabilitation significantly increased overall adherence and, together with exercise and nutrition, yielded superior functional outcomes (40). Thus, psychological care functions both as an independent therapeutic domain and as a catalyst, increasing engagement and persistence to amplify the effects of other components.

Collectively, the principal advantage of multimodal prehabilitation lies in reciprocal gains among its elements: exercise provides the physiological stimulus, nutrition supplies the anabolic substrate, and psychological support secures adherence and execution. The dual improvements in function and efficiency observed in the present study likely reflect the integrated impact of these multichannel, multi-target interactions.

### 4.3 Prospects for prehabilitation intervention intensity and patient adherence

A substantial body of randomized trials and systematic reviews indicates a characteristic dose–response relationship for both exercise and IMT. Andersson et al. reported that IMT sustained for at least two weeks with daily sessions of ≥15 min is associated with a significant reduction in postoperative pulmonary complications, whereas protocols with insufficient intensity or duration yield inconsistent effects (41). Beyond clinical endpoints, sub-therapeutic loads are unlikely to induce meaningful respiratory muscle remodeling or increases in diaphragmatic thickness; gains tend to be confined to endurance rather than genuine strength enhancement. Similar principles apply to aerobic conditioning. As shown by Vasić et al., maintaining training at 60%–80% of the age-predicted maximal heart rate produces marked improvements in oxygen-uptake thresholds and exercise tolerance, while lower intensities elicit only limited metabolic adaptation (42). Insufficient dosing therefore attenuates durable benefits on cardiorespiratory reserve and performance. In the present study, higher training frequency and on-site supervision enabled clinically meaningful improvement within a short inpatient window, suggesting that “short-course” regimens can be effective when intensity and frequency compensate for limited duration. Looking ahead, standardized prescriptions grounded in the FITT framework (frequency, intensity, time, type) are needed, alongside stratified evaluations to identify optimal intensity combinations for high-risk subgroups (e.g., patients with malnutrition or sarcopenia).

Adherence represents the second critical determinant of effectiveness. Even well-designed protocols underperform if patients do not execute them as intended. Several trials have shown greater variability—and often smaller effects—on functional outcomes and length of stay with fully home-based programs, in part due to inadequate self-monitoring, sub-threshold training loads, or declining frequency over time (43, 44). In contrast, Bousquet-Dion et al. demonstrated that supervised, multimodal prehabilitation improves adherence and delivers more consistent functional gains than home-based exercise alone (45). In the current study, face-to-face inpatient guidance minimized execution bias and safeguarded intervention quality; nevertheless, limited hospitalization means that sustained post-discharge engagement is frequently lacking. The emergence of digital health and telerehabilitation makes hybrid supervision increasingly feasible: initiating at high intensity in hospital, then transitioning to remote monitoring, wearable-based tracking, and behavioral interventions can preserve training quality while containing resource use. Early implementations suggest that remote monitoring coupled with timely feedback substantially improves adherence; in large, multicenter settings, such hybrid models may offer a pragmatic balance between feasibility and effectiveness (46).

## 5 Conclusion

The study demonstrates that a modified multi-modal prehabilitation program is feasible and effective for improving preoperative physical function and postoperative recovery in Chinese patients undergoing colorectal cancer surgery. This approach not only enhances the 6MWT outcomes but also reduces postoperative hospital stay and accelerates recovery milestones, without increasing postoperative complications. These findings support the implementation of prehabilitation as an integral part of colorectal cancer surgical care to optimize patient outcomes.

## Data Availability

The raw data supporting the conclusions of this article will be made available by the authors, without undue reservation.
